# Synthesis and Evaluation of a Novel Cassava-Based Starbon-Type Mesoporous Catalyst

**DOI:** 10.1021/acsomega.6c03006

**Published:** 2026-08-19

**Authors:** Milena A. Zabala, Suranjana Bose, Laura J. Panqueba, Alvaro Orjuela, James H. Clark

**Affiliations:** † Department of Chemical and Environmental Engineering, 28021 Universidad Nacional de Colombia, 111321 Bogotá D.C., Colombia; ‡ Green Chemistry Centre of Excellence, Department of Chemistry, 8748 University of York, YO10 5DD York, U.K.

## Abstract

Mesoporous materials are well suited as supports for heterogeneous catalysis because they facilitate the access and mobility of large molecules, thereby improving yields across a range of chemical reactions. Among these, mesoporous carbons can be advantageously synthesized from biobased sources and further functionalized for use as acid catalysts in esterification reactions. This work focuses on the production of an esterification catalyst based on starch-derived mesoporous carbon (Starbon), using cassava starch as the raw material, and compares its performance with that of a conventional corn starch-based (Hylon VII) Starbon. The catalysts were prepared via gelatinization in water, retrogradation, solvent exchange with t-butanol, freeze-drying, and carbonization of expanded starch at 450 °C, followed by sulfonation with sulfuric acid. The resulting cassava-derived Starbon exhibited a surface area of 263 m^2^/g and a mean pore diameter of 8.1 nm, comparable to those of commercial corn-based material (271 m^2^/g and 14.5 nm). The catalysts were evaluated in the batch esterification of stearic acid with isopropyl alcohol, and their performance was compared with that of several commercial homogeneous (H_2_SO_4_, *p*-toluenesulfonic acid, methanesulfonic acid) and heterogeneous (Amberlyst 15, Amberlyst 35) catalysts. Despite performing below homogeneous catalysts, cassava-based Starbon outperformed all heterogeneous catalysts, reaching an apparent turnover frequency of 0.2 mol·s^–1^·H^+^ Equiv^–1^ and a final conversion of 24% after 5 h. Postreaction acidity measurements indicated that the catalyst is chemically stable; however, it suffers from mechanical instability. This is likely due to the high amylopectin content in cassava starch, which hinders the formation of a robust and stable porous structure.

## Graphical abstract

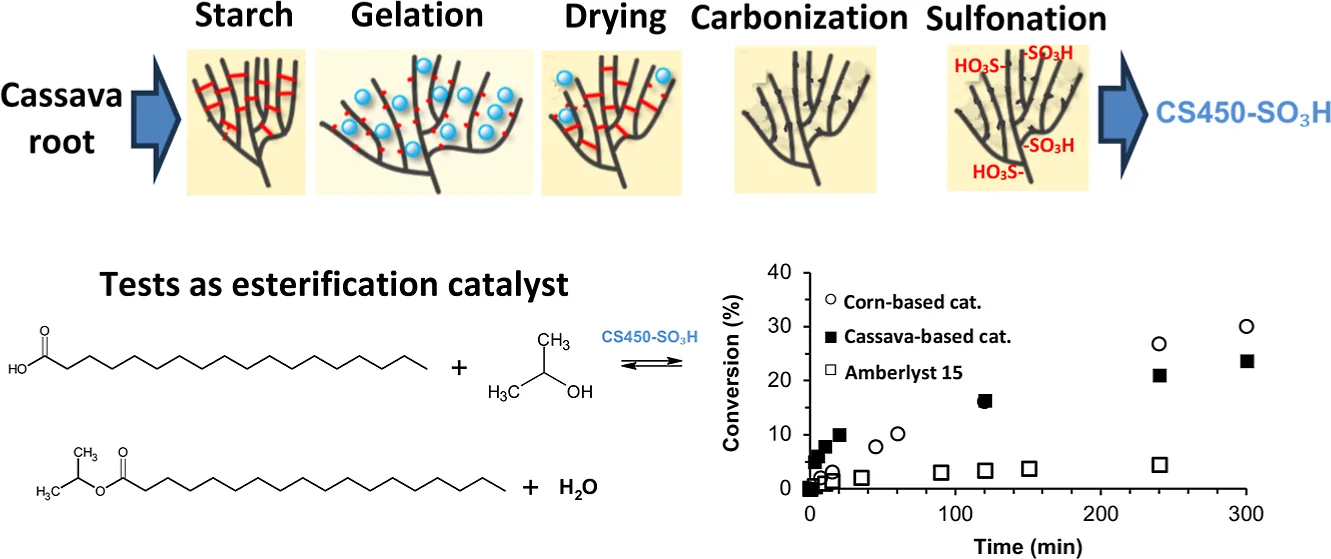

## 1 Introduction

Fatty acid esters (FAEs) are key oleochemicals widely used in the manufacture of food and personal care products, pharmaceuticals, cosmetics, solvents, and biofuels, among many other applications. The global FAE market is currently valued at approximately 2.8 billion per year and is projected to reach 3.8 billion by 2033.^1^ In general, FAEs are produced via Fischer esterification of fatty acids with various alcohols using homogeneous acid catalysts such as sulfuric acid, *p*-toluenesulfonic acid (PTSA), or methanesulfonic acid (MSA).^2–5^ To meet commercial specifications, complete removal of the homogeneous catalyst is required, which typically involves intensive downstream separation, neutralization, and significant waste generation.^6^ Moreover, homogeneous catalysts can promote equipment corrosion and undesired side reactions, including alcohol etherification as well as dehydration and oxidation of organic compounds, leading to product darkening.^6^ Besides, the presence of sulfonic acid esters in FAEs used as cosmetics ingredients has been recently confirmed.^7,8^ These are considered potentially genotoxic byproducts and they are produced when using strong homogeneous sulfonic acid catalysts.^7–9^ As an alternative, commercial heterogeneous acid catalysts, such as ion-exchange resins, have been evaluated for FAE production.^10–13^ Because the kinetic diameter of FAEs is approximately 0.8–1.5 nm,^14^ mesoporous macroreticular cross-linked resins (e.g., Amberlyst 15) are generally preferred due to their large pore sizes (28–30 nm).^15,16^ This mesoporosity enhances molecular mobility within the polymer matrix and improves accessibility to active sites. However, polymeric acid resins exhibit high water uptake, which is disadvantageous for FAE synthesis, as esterification is equilibrium-limited.^10,12^ Additionally, although these catalysts enable easier recovery and reuse, most ion-exchange resins can only operate at temperatures up to 120–150 °C to prevent degradation of the polymer matrix and subsequent deactivation, thereby limiting achievable reaction rates. This is a key point because the intensified production of fatty esters has been addressed via reactive distillation, and there is need to operate under high bubble temperatures to ensure high reaction rates under acid catalysts.^13,17–23^

To overcome these operating limitations, heterogeneous catalysts have been increasingly engineered with tailored morphologies and defined porous structures. In particular, biobased materials have emerged as flexible, functionalized feedstocks for the synthesis of mesoporous catalysts. By exploiting the intrinsic characteristics of various biobased sources (e.g., seed shells, fibers, husks, bones, seashells, and polysaccharides), or by processing them into designed porous matrices, it is possible to obtain solids with morphologies well suited to a range of catalytic applications. Beyond enhancing activity and selectivity, the use of biobased precursors helps reduce the life-cycle impacts of catalysts and mitigates challenges associated with spent catalyst disposal, thereby improving overall sustainability metrics. Among the wide range of biobased catalysts, acid-activated carbons have been successfully synthesized and applied to the esterification of various carboxylic acids.^24–31^ In addition to their superior thermal stability, their hydrophobic nature allows them to achieve high conversions even in the presence of water in the reaction medium.^25,28,29^ Notably, certain activated carbons have demonstrated excellent catalytic performance in the production of fatty acid methyl esters, delivering high yields within remarkably short reaction times, typically under 0.5 h.^32^ An alternative approach involved the use of biochar-based catalysts from marine macroalgae which were subjected to thermochemical treatment (180–900 °C), followed by activation via physical methods (e.g., steam or CO_2_) or chemical treatments, including alkaline, acidic, and oxidative processes. The resulting catalysts have shown significant yields in fatty ester production, with reaction times ranging from 1 to 5 h, depending on the synthesis route and operating conditions.^33^ Despite these promising results, further development of carbon-based catalyst synthesis is necessary to achieve more sustainable processes, particularly considering their use as internals in reactive distillation columns.^34,35^ Consequently, several alternative and more sustainable synthesis strategies have been explored. For example, biochar catalysts derived from lignin-based monomers via low-temperature thermochemical treatment (∼80 °C) have shown promising yields; however, reaction times remain relatively long, on the order of 6 h.^36^

Given the relatively low reaction rates, the synthesis of FAEs using carbon-based catalysts can be enhanced through process intensification strategies, such as sonication and microwave irradiation. For example, the reaction time for producing canola-based biodiesel using activated carbon derived from calcined lime has been reduced to only 15 min under microwave irradiation.^37^ Alternatively, as summarized in Table 1, fatty acid esterification can be improved by employing tailored mesoporous structures. Mesoporous activated carbons have been prepared via controlled carbonization of organic precursors that exploit their inherent porous architecture, through carbonization of preformed structures, or by using sacrificial inorganic templates (e.g., mesoporous silica or alumina). In the latter approach, high-temperature carbonization is typically required to enable the removal of the inorganic template. However, this process not only destroys the structural complexity and entropy encoded in the template but also relies on hazardous reagents, such as hydrofluoric acid, and generates substantial waste, limiting its industrial sustainability.^27,28^

**Table 1:** Reported Studies on the Synthesis of Biobased Mesoporous Carbon Catalysts for the Esterification of Fatty Acids^c^ ^d^

biobased source	synthesis conditions	area m^2^/g	*D*_P_ ^a^ (nm)	*V*_P_ ^b^ (cm^3^/g)	activation	acidity (m eqiuv H^+^/g)	fatty acid esterification case	ref
sucrose	impregnation of mesoporous silica, calcination, activation, template removal with HF	808	3.9	0.47	sulfonation with H_2_SO_4_	2.3	oleic acid/methanol 1:10 mol, cat 0.7% wt. 353 K, X—60%, 10h	^38^
		318–805	2.8–6.8	0.04–0.25	atachment of benzenesulfonic acid	1.7–2.6	oleic acid/methanol 1:56 mol, cat 2.9% wt. 338 K, X—90%, 2.5h	^39^
starch	sulfonation of Starbon 300 obtained from Hylon VII	66–77	11–15	-	sulfonation with H_2_SO_4_	1.1–2.3	oleic acid/ethanol 1:10 mol, cat 1.3% wt. 353 K, X—90%, 10h	^40^
	carbonization and sulfonation of commercial starch	12.40	44	0.51	sulfonation with H_2_SO_4_	6.97 SO_3_H	palm fatty acids/methanol 1:10 mol, Cat 2% wt. 348 K, X—94.6%, 3h	^41^
cleargum starch	gelling, drying, carbonization, activation	32	30	0.16	sulfonation with H_2_SO_4_	0.82	lauric acid/methanol 1:15 mol, cat 3% wt. 343 K, X—77%, 12h	^42^
-	impregnation of mesoporous γ-alumina, calcination, activation, template removal with HF	493–616	3.3–3.8	0.35–0.44	phosphorylation with H_3_PO_4_	0.56–1.12	oleic acid/methanol 1:15 mol, cat 1.8% wt. 353 K, X—70%, 5h	^43^
pongamia galabra seed cake	activation-carbonization, sulfonation	483	4.7	0.58	sulfonation with 4-benzene-diazonium sulfonate	3.62	acid oil/methanol, 1:40 mol, cat 0.9% wt. 393 K, X - 93%, 2h	^44^
bamboo	carbonization, activation	226	10–20	0.12	attachment of sulfanilic acid	1.69	oleic acid/ethanol 1:7 mol, cat 5.6% wt. 358 K, X—96%, 3h	^45^
palm biomass	hydrothermal Carbonization, impregnation	273–639	4	0.2–0.5	impregnation with Cu(NO_3_)_2_	1.6–1.8	acidic used cooking oil: methanol 12:1, cat 5% wt. 343 K, X—96%, 2h	^46^
corn cob	carbonization, sulfonation	398	3.1–4.8	1.39	sulfonation with 1,3-propanesulfonate	1.42	oleic acid/ethanol 1:12 mol, cat 0.7% wt. 353 K, X > 94%, 2h	^47^

a Average pore diameter.

b Pore volume.

c Cat—catalyst loading with respect to total weight.

d X—conversion.

To address these challenges, template-free mesoporous carbons have been developed through the preformation and subsequent carbonization of organic aerogels, xerogels, and cryogels. The use of preformed mesoporous gel structures has enabled the development of catalysts with improved mechanical resistance and high surface area per unit volume.^48–50^ Although these materials were initially derived from fossil-based resources,^50^ they have since been successfully produced from storage and structural polysaccharides such as starch, glycogen, cellulose, chitin, and pectin. These biobased materials offer several advantages: they are widely available at low cost, contain functional groups suitable to further activation, and readily form stable, structured gels in aqueous media.^54^ As a result, starch-based mesoporous activated carbons have been commercialized in recent years under the trade name Starbon.^51–53^ These materials have been evaluated in various acid-catalyzed reactions, demonstrating high stability, reusability, and excellent performance in terms of reaction rates, conversions, and selectivity.^25,46,51,55^ Furthermore, Starbon materials can be produced from second-generation starch sources (e.g., food waste), thereby supporting circular economy principles in their synthesis. This aspect is particularly relevant for countries such as Colombia, where nearly 30% (approximately 1.5 Mt) of the annual production of starch-rich roots and tubers is discarded due to failure to meet quality standards.^56^ In addition, substantial starchy waste streams are generated in agro-industrial processes, especially during the processing of cassava (*Manihot esculenta*).^57,58^

It is worth mentioning that even though the use of carbonization, solvents for retrogradation, and sulfonation agents (e.g., sulfuric acid, oleum, SO3) is not completely aligned with green chemistry principles, such processes employ waste biomass, they are cost-effective, and readily available for the activation of Starbon catalysts.^24,25,59^ In addition to using waste biomass and to minimize their potential environmental impacts, gases from carbonization can be integrated in a cogeneration or heating system, carbonization could be done via a microwave-assisted process, solvents can be recovered during drying, and the sulfonating agents used during the activation process could be recycled and reused to extinction in the preparation of the sulfonated materials. In this context, this work explores the synthesis of a sulfonated starbon catalyst derived from cassava starch as an alternative feedstock to conventional corn-based starbon. The catalyst is intended for the esterification of fatty acids with particular emphasis on the production of isopropyl FAEs. The synthesis process involves starch gelation and retrogradation in aqueous media, followed by solvent exchange from water to an organic phase, solvent removal via freeze-drying, and controlled carbonization. The resulting biobased carbon was subsequently activated through sulfonation with sulfuric acid. The obtained materials are then characterized and evaluated as catalysts in the esterification of stearic acid with isopropanol. For benchmarking purposes, a conventional corn-based starch (Hylon VII) was also used to prepare a Starbon-type esterification catalyst. In addition, to assess relative activity and enable comparison, a set of commercial homogeneous (H_2_SO_4_, *p*-toluenesulfonic acid, and methanesulfonic acid) and heterogeneous (Lewatit K2629 and Amberlyst 15, 35, and 70) catalysts was evaluated under equivalent acid loads. The cassava-based catalysts developed in this study are expected to have potential applicability beyond the present system, offering a promising route toward more-sustainable catalytic materials.

## 2 Materials and Methods

### 2.1 Materials

Corn starch was obtained from an industrial supplier (Hylon VII, Ingredion) and contained 68–70 wt % amylose and approximately 30 wt % amylopectin. Refined cassava starch was a commercial retail food product (>87 wt % starch, Yucarina, Unilever) obtained from a local grocery store and was used without further purification. Deionized water for the gelation process was produced in the laboratory. *Tert*-butyl alcohol (99.7 wt %, PanReac AppliChem ITW Reagents) was used for solvent exchange, while *p*-toluenesulfonic acid monohydrate (97 wt %, Fisher Scientific) was employed both for doping the material prior to carbonization and as a homogeneous catalyst. Triple-pressed stearic acid (95 wt %, Legaquímicos) and isopropanol (99.90 wt %, J.T. Baker) were used in the esterification experiments. Solutions of 0.1 and 0.25 M NaOH (99 wt %, PanReac AppliChem ITW Reagents) were prepared for ion-exchange capacity measurements and acid value (AV) monitoring, respectively. Ethanol (99.5 wt %, PanReac AppliChem ITW Reagents) and toluene (99.5 wt %, PanReac AppliChem ITW Reagents) were used as solvents during AV determination. Sulfuric acid (98 wt %, PanReac AppliChem ITW Reagents), *p*-toluenesulfonic acid monohydrate (97 wt %, Thermo Fisher Scientific), and methanesulfonic acid (70 wt %, Fisher Scientific) were also used as homogeneous catalysts in esterification reactions. Commercial heterogeneous catalysts used for comparative evaluation included Amberlyst 15, Amberlyst 35, and Amberlyst 70 (Dow Chemical),^60–62^ as well as Lewatit K 2629 (Lanxess).^63^ Selected physicochemical properties of these heterogeneous catalysts are summarized in Table 2.

**Table 2:** Properties of the Heterogeneous Catalysts Tested in the Esterification of Stearic Acid with Isopropanol

catalyst	amberlyst 15WET^60^	amberlyst 35WET^61^	amberlyst 70 ^62^	lewatit K2629 ^63^
moisture (%)	52–57	51–57	53–59	50–55
BET surface area (m^2^/g)	53	50	36	40
average pore diameter (nm)	30	30	22	40
total pore volume (cm^3^/g)	0.40	0.35	not reported	0.30

### 2.2 Methods

#### 2.2.1 Preparation of Starbon-type Materials

The commercial cassava starch used as a precursor was first characterized to determine its amylose and amylopectin content using an enzymatic method (K-AMYL 06/18).^64^ Subsequently, both corn- and cassava-based mesoporous carbons were synthesized following procedures reported in the literature for conventional Starbon materials.^25,27,42,48,51,59^ Briefly, a defined amount of starch was dispersed in deionized water at a 1:10 mass ratio, and the mixture was heated at 90 °C under atmospheric pressure for 1 h. The resulting gel was then retrograded at 5 °C for 48 h. Solvent exchange was subsequently carried out by adding *tert*-butyl alcohol to reach 30 wt % of the mixture, along with *p*-toluenesulfonic acid (5 wt %) as a doping agent. The material was then freeze-dried at −107 °C and 1 mbar to obtain an expanded starch. The dried material was carbonized at 450 °C using the following temperature program: heating from 25 to 170 °C at 10 K·min^–1^, from 170 to 400 °C at 5 K·min^–1^, and from 400 to 450 °C at 1 K·min^–1^. Carbonization was performed under a nitrogen atmosphere to develop appropriate hydrophobic/hydrophilic characteristics for esterification reactions.^51^ In this work, the resulting Starbon-type materials are denoted as S450 for the corn-based carbon and CS450 for the cassava-derived carbon.

#### 2.2.2 Functionalization

The obtained Starbon-type materials were sulfonated using concentrated sulfuric acid (99.99 wt % purity) at an acid-to-carbon ratio of 10 mL g^–1^, at 80 °C for 8 h. The resulting sulfonated solids were thoroughly washed with distilled water until no sulfate ions were detected in the effluent, as confirmed by a barium chloride test. After washing, the materials were subjected to methanol reflux overnight (5 mL methanol per g of Starbon) to remove physiosorbed sulfuric acid and residual oligomeric species. Finally, the samples were dried overnight in an oven at 100 °C. The resulting activated carbons are denoted as S450–SO_3_H for the corn-based material and CS450–SO_3_H for the cassava-derived material. The overall preparation and functionalization procedure is schematically illustrated in Figure 1.

**Figure 1:**
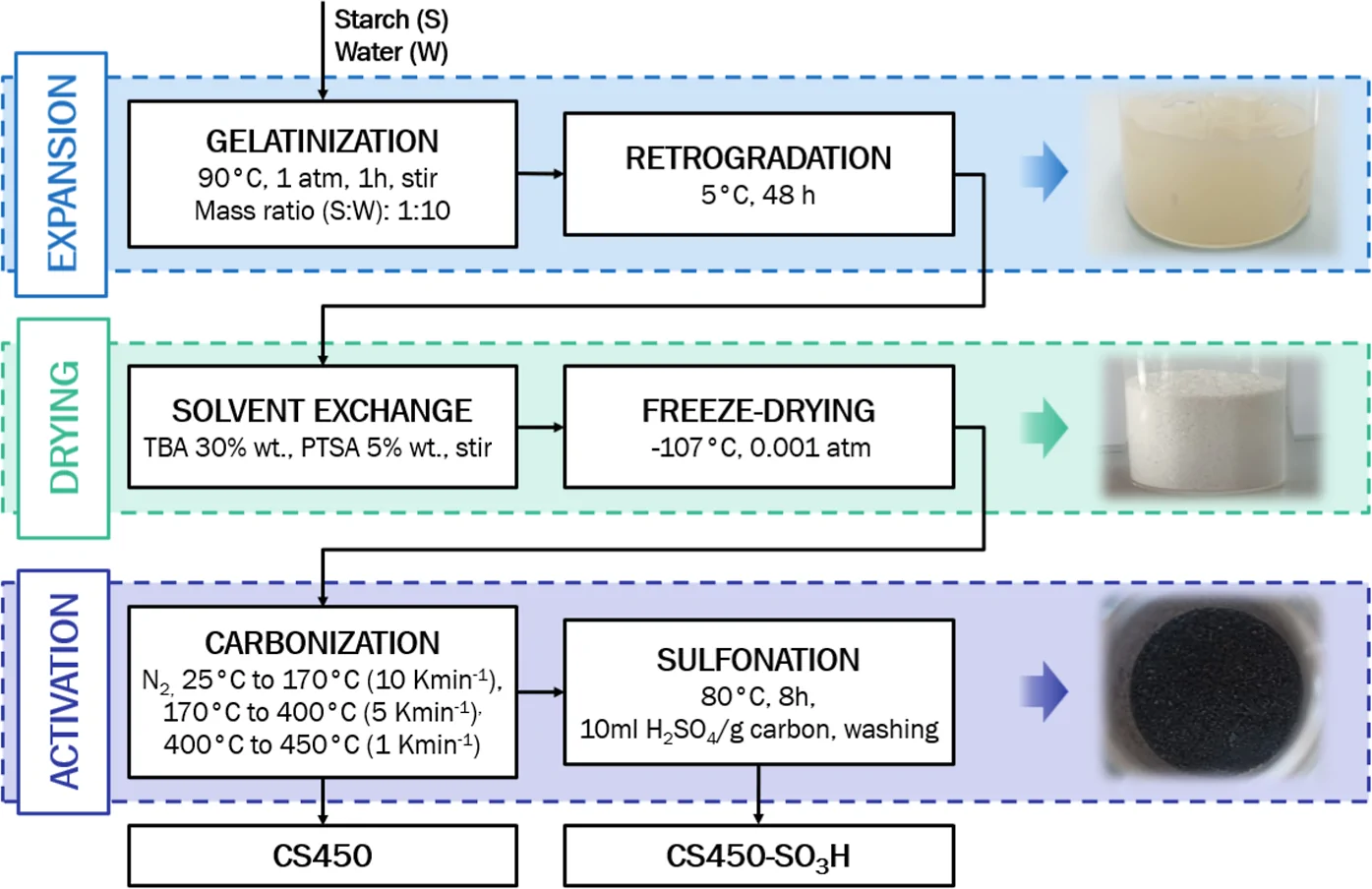
Schematic description of the methodology used in catalyst preparation.

#### 2.2.3 Characterization

The ion-exchange capacities of the sulfonated Starbon materials and the other heterogeneous catalysts were determined by back-titration. In brief, 1 g of the dried catalyst was added to a 0.1 M NaOH solution and stirred for 5 h at room temperature. The suspension was then filtered to remove the solid, and the remaining NaOH in the filtrate was titrated to determine its final concentration. The ion-exchange capacity of the sulfonated Starbon samples was also evaluated after use in esterification reactions. In this case, the catalysts were recovered from the reaction mixture, washed with anhydrous ethanol, and dried to a constant weight prior to analysis.

The surface area and porosity of the sulfonated Starbon materials were determined from nitrogen adsorption–desorption isotherms measured at −195.5 °C using an ASAP 2020 V4.02 volumetric adsorption analyzer (Micromeritics). Prior to analysis, the samples were degassed for 24 h. Data were interpreted using the multipoint Brunauer–Emmett–Teller (BET) method, while pore size distributions were obtained using the Barrett–Joyner–Halenda (BJH) model. The morphology and pore structure of the materials were examined by scanning electron microscopy (SEM) using a FEI Quanta 200 instrument. Samples were sputter-coated with a thin gold layer prior to imaging, and micrographs were acquired at different magnifications (5000× and 10,000×).

The nature of the organic functional groups in the carbon-based materials was analyzed by Fourier-transform infrared (FT-IR) spectroscopy using a PerkinElmer FT-IR 1760 spectrometer, with spectra recorded over the range 4000–500 cm^–1^. Thermal stability was evaluated by thermogravimetric analysis (TGA) using a Mettler Toledo TGA 1/SF/1100/268 instrument. Approximately 4.5 mg of the sample was analyzed over a temperature range of 30–1000 °C at a heating rate of 10 °C min^–1^ under a nitrogen atmosphere. Weight changes at low temperatures during TGA can also provide insights regarding water retention of the materials.

#### 2.2.4 Esterification Tests

Esterification of stearic acid with isopropyl alcohol (1:2 molar ratio) was conducted at 70 °C and atmospheric pressure for 5 h. The reaction was carried out in a 200 mL jacketed three-neck glass reactor operated under total reflux, equipped with a 200 mm 19/26 Graham condenser and mechanically stirred using a Teflon-coated magnetic stirrer at 450 rpm. Preliminary experiments were performed using H_2_SO_4_ as a benchmark catalyst, as it is the most commonly employed homogeneous catalyst for industrial esterification, typically used at loadings of 0.5–1 wt % relative to stearic acid. Subsequently, all catalytic tests were conducted under an equivalent acid loading corresponding to 1 wt % H_2_SO_4_. In the case of solid catalysts, acid equivalents were calculated based on their measured ion-exchange capacity. Prior to use, commercial ion-exchange resins were pretreated by washing with ethanol in a stirred glass vessel for 2 h, using an ethanol-to-resin volumetric ratio of 4:1. This washing step was repeated three times. The resins were then rinsed with deionized water, filtered, and dried under vacuum at 60 °C to constant weight. The dried materials were stored in sealed, light-protected containers in a desiccator until use.

Reaction progress was monitored by withdrawing samples at different time intervals using a preheated pipet to prevent stearic acid solidification during sampling. The collected samples were analyzed by determining the AV according to ISO 660 E.^65^ Conversion (*X*) as a function of time was calculated using eq 1.

1 $$X=\frac{{AV}_{0}-AV}{AV_{0}}$$

Here, *AV*_0_ is the initial *AV* of the triple pressed stearic acid (207.11 mg KOH/g sample) and *AV* is the acid value of the reaction sample on an alcohol- and water-free basis.

## 3 Results and Discussion

### 3.1 Characterization of Biobased Activated Carbons

After characterization of the commercial cassava starch used as feedstock, it was found to contain 20.7 wt % amylose and 79.3 wt % amylopectin. This composition represents a challenge, as high-amylose starches are generally preferred for Starbon synthesis.^24,25,66^ Nevertheless, it has been reported that low-amylose starches can also be used as precursors, although they typically yield materials with lower pore diameters and surface areas.^66,67^ The nitrogen adsorption–desorption isotherms for the corn-based (S450) and cassava-based (CS450) carbons are shown in Figure 2, while the complete surface and porosity characterization data are summarized in Table 3. As observed, the isotherms of both Starbon-type materials can be classified as type I, with H3-type hysteresis loops. This behavior is indicative of a mixed micro- and mesoporous structure with slit-shaped pores, which is characteristic of this class of activated carbons.^68^

**Figure 2:**
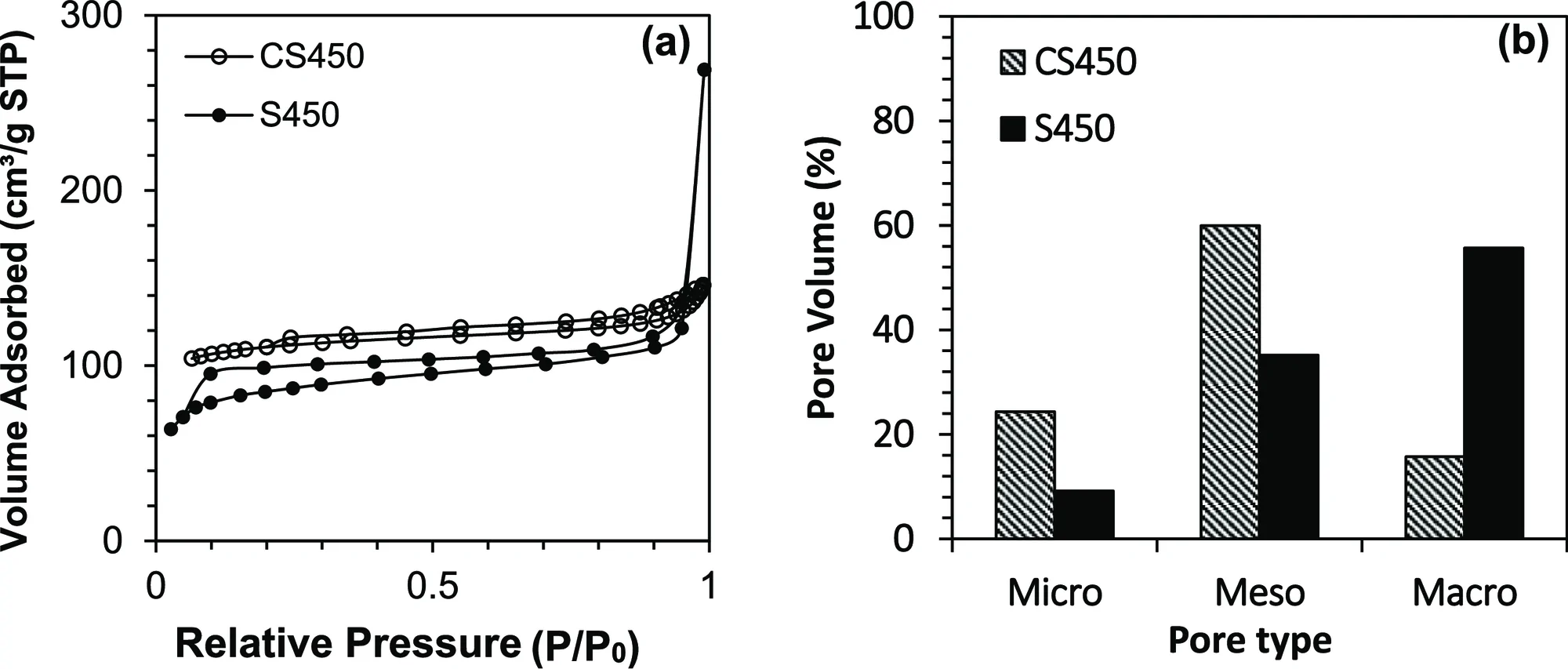
Nitrogen adsorption isotherms (a) and porosity histograms (b) for sulfonated cassava-based (CS450-SO_3_H) and corn-based (S450-SO_3_H) carbon materials.

**Table 3:** Surface Area and Porosity of Assessed Sulfonated Corn-Based (S450-SO_3_H) and Cassava-Based (CS450-SO_3_H) Carbons Catalysts

property	S450-SO_3_H	CS450-SO_3_H
BET surface area (m^2^/g)	271	263
BJH average pore diameter (nm)	14.5	8.1
micropore content (cm^3^/g)	0.037	0.053
mesopore content (cm^3^/g)	0.143	0.132
macropore content (cm^3^/g)	0.230	0.035
total pore volume (cm^3^/g)	0.410	0.22

As expected from the amylose content and according to the porosity histogram of Figure 2b, Cassava Starbon CS450-SO3H exhibited a wider range of porosity with a higher content of mesopores (60%) and micropores (24%). In comparison, corn-based S450-SO3H is mainly composed of meso- and macropores. According to the BJH analysis, the average pore width of the CS450-SO3H was 8.1 nm, and the corresponding BET surface area was 263.1 m^2^/g. Comparatively, S450-SO3H had an average pore diameter of 14.5 nm and a BET surface area of 271.1 m^2^/g. This was consistent with a larger fraction of macropores in the structure of S450-SO3H and with the previously observed morphology of low-amylose-based Starbons.^66,67^

The morphology of CS450–SO_3_H and S450–SO_3_H particles is shown in the SEM micrographs in Figure 3. As observed, for the corn-based material (Figure 3a,b), the granular structure of the native starch is largely preserved in the final catalyst. In contrast, for the cassava-based Starbon (Figure 3c,d), the granular morphology is significantly degraded. Considering the scaffolding role of amylose, the lower amylose content in cassava starch likely results in insufficient structural support, leading to partial collapse of the gel network during solvent exchange and drying. Instead of retaining a granular form, this collapse produces a filamentous and sponge-like structure with higher surface area and increased microporosity. The subsequent sulfonation step may further contribute to structural weakening and partial degradation of this already fragile framework. Accordingly, CS450–SO_3_H is expected to exhibit lower mechanical strength and reduced recoverability after reaction. Nevertheless, shall it demonstrate high catalytic activity in esterification, strategies may be required to enhance its structural stability such as optimization of thermal treatments^69,70^ or cross-linking prior carbonization.^71–75^

**Figure 3:**
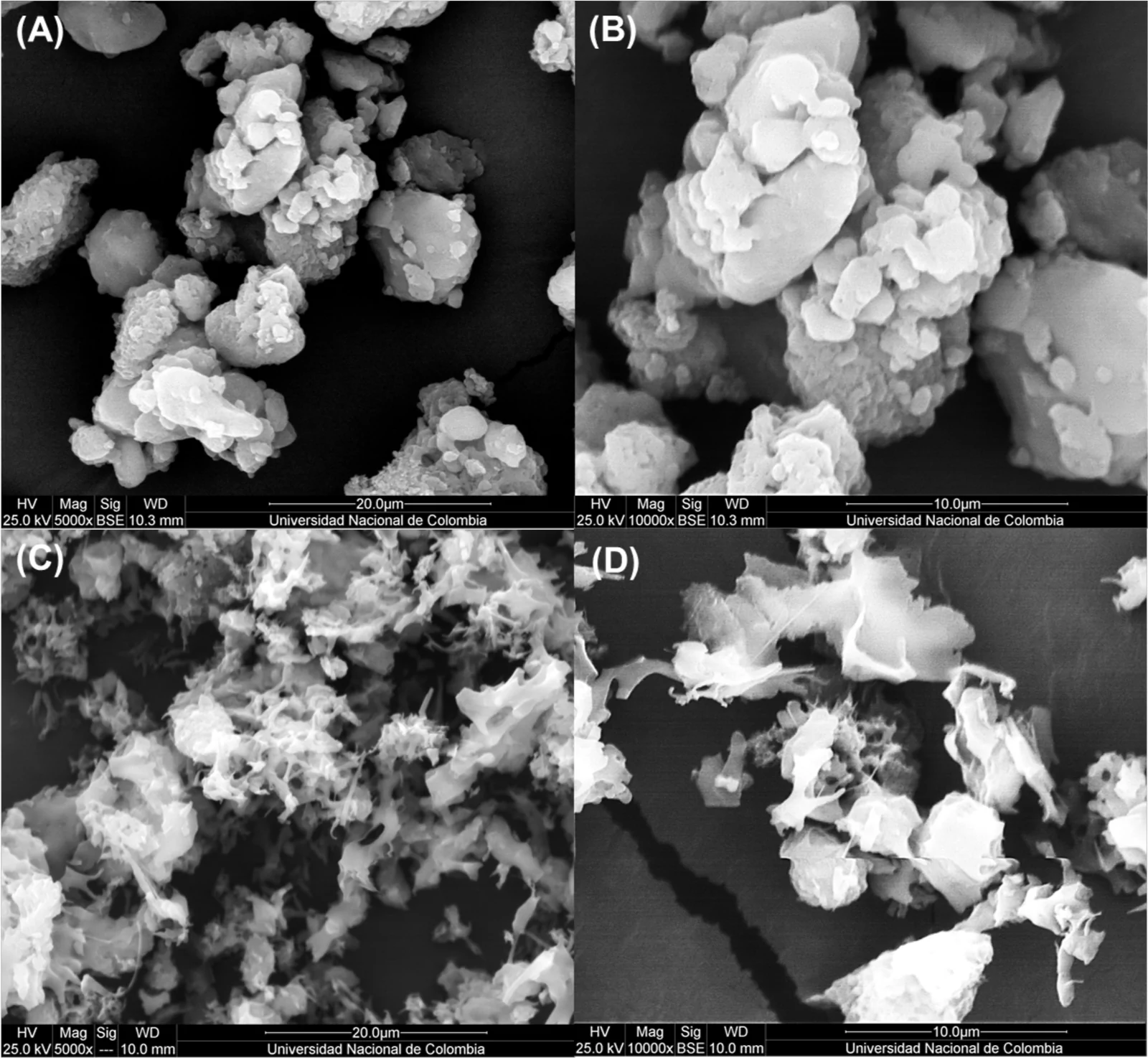
SEM micrographs obtained at different magnifications (5000× left and 10000× right) for S450-SO_3_H (A,B) and CS450-SO_3_H (C,D).

The FT-IR spectra of CS450–SO_3_H and S450–SO_3_H are shown in Figure 4. The spectrum of the nonsulfonated cassava-based material (CS450) is also included to highlight the chemical changes in the structure upon sulfonation. In all samples, the broad band in the 3200–3600 cm^–1^ region is attributed to O–H stretching vibrations,^76^ arising from hydroxyl groups in the original saccharide structure, carboxylic groups formed during carbonization, phenolic hydroxyls generated in the carbon framework, and adsorbed water.^77^ Notably, both CS450–SO_3_H and S450–SO_3_H exhibit a characteristic peak at 1024 cm^–1^, assigned to S–O stretching vibrations of −SO_3_^–^ groups,^76^ confirming successful sulfonation of the materials. This is further supported by the increased in the region around 1150 cm^–1^, associated with S=O stretching vibrations. Bands corresponding to C=O and C=C stretching vibrations are observed at approximately 1705 cm^–1^ and 1600 cm^–1^, respectively,^76^ in all three materials. In addition, peaks at 1456 cm^–1^ are attributed to C–C skeletal vibrations of aromatic structures.^76^ Overall, these features are consistent with those typically reported for sulfonated activated carbons.

**Figure 4:**
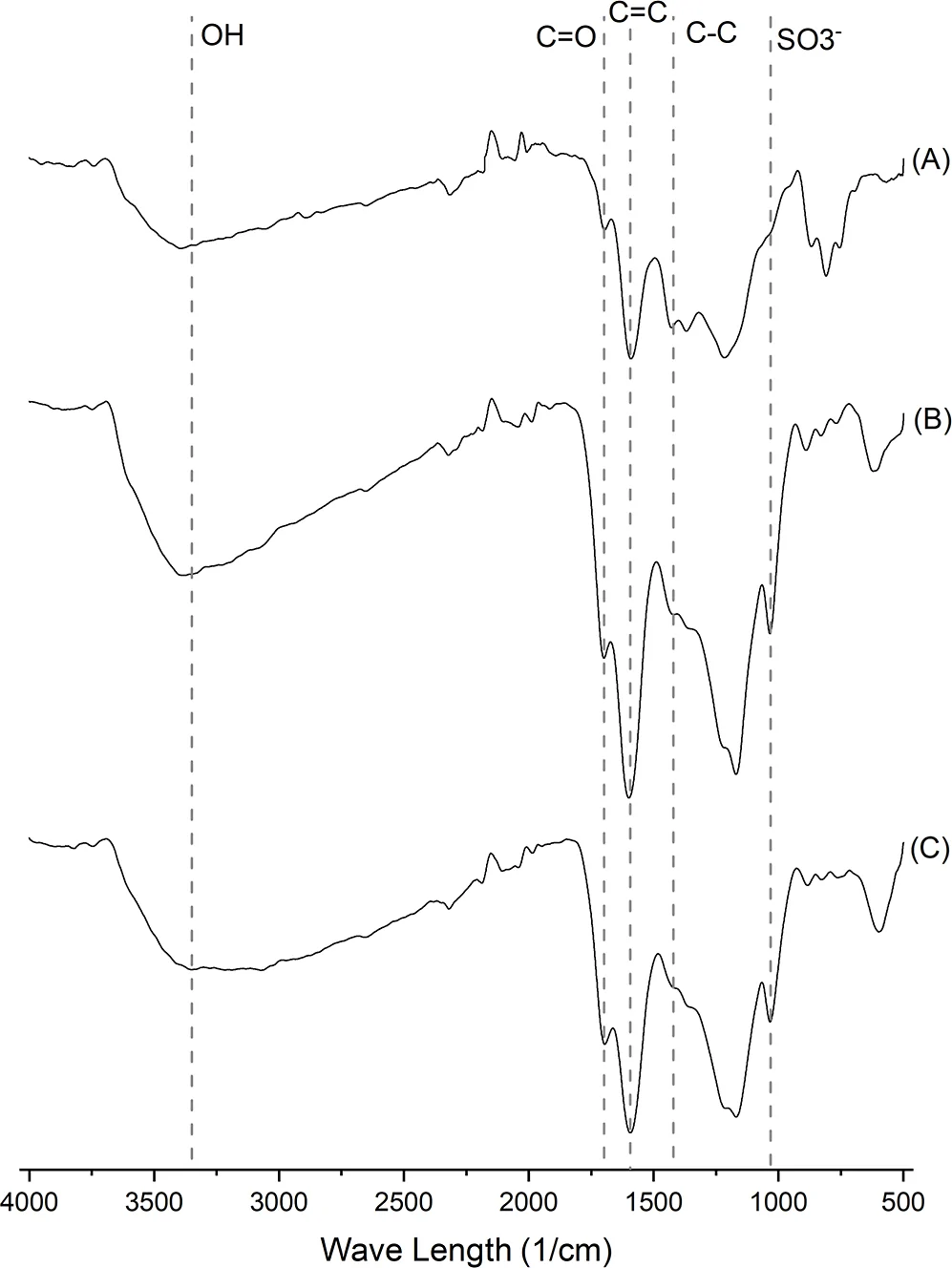
FT-IR spectra for CS450 (A), CS450-SO3H (B), and S450-SO3H (C).

The ion exchange capacities of the different catalysts are reported in Table 4. Although the values for commercial ion-exchange resins are provided in their technical data sheets, they were also independently measured on a dry basis for validation and comparison. As shown, sulfonation of the starch-derived materials was effective, yielding acidity values comparable to those of the commercial ion-exchange resins. However, given the collapsed gel structure of the cassava-based material, the anchoring of sulfonic groups within the mesoporous network is expected to be less stable. Consequently, partial catalyst loss due to attrition during the reaction cannot be ruled out.

**Table 4:** Ion Exchange Capacity of Assessed Heterogeneous Catalysts

catalyst	exchange capacity—dry basis (equiv H^+^/kg)
amberlyst 15	4.56
amberlyst 35	3.85
amberlyst 70	2.78
lewatit K2629	4.33
S450-SO_3_H	2.31
CS450-SO_3_H	3.60

### 3.2 Reaction Tests

As a benchmark, the kinetic profiles for the esterification of stearic acid with isopropanol using H_2_SO_4_ as catalyst are presented in Figure 5. For comparison purposes, experiments were conducted at different catalyst loadings, namely 0% (self-catalyzed reaction), 0.5 wt %, and 1 wt % relative to stearic acid. As observed, the catalyzed reaction reaches a conversion of approximately 63% after 5 h. In contrast, the uncatalyzed reaction remains below 4% conversion over the same period. These results are consistent with previous reports on the esterification of stearic acid with monohydric alcohols.^4,78–82^ In this case, the rates of reaction are lower compared to other alcohols because isopropanol is a secondary alcohol and the hydroxyl group is less reactive compared with primary groups in other monohydric alcohols.

**Figure 5:**
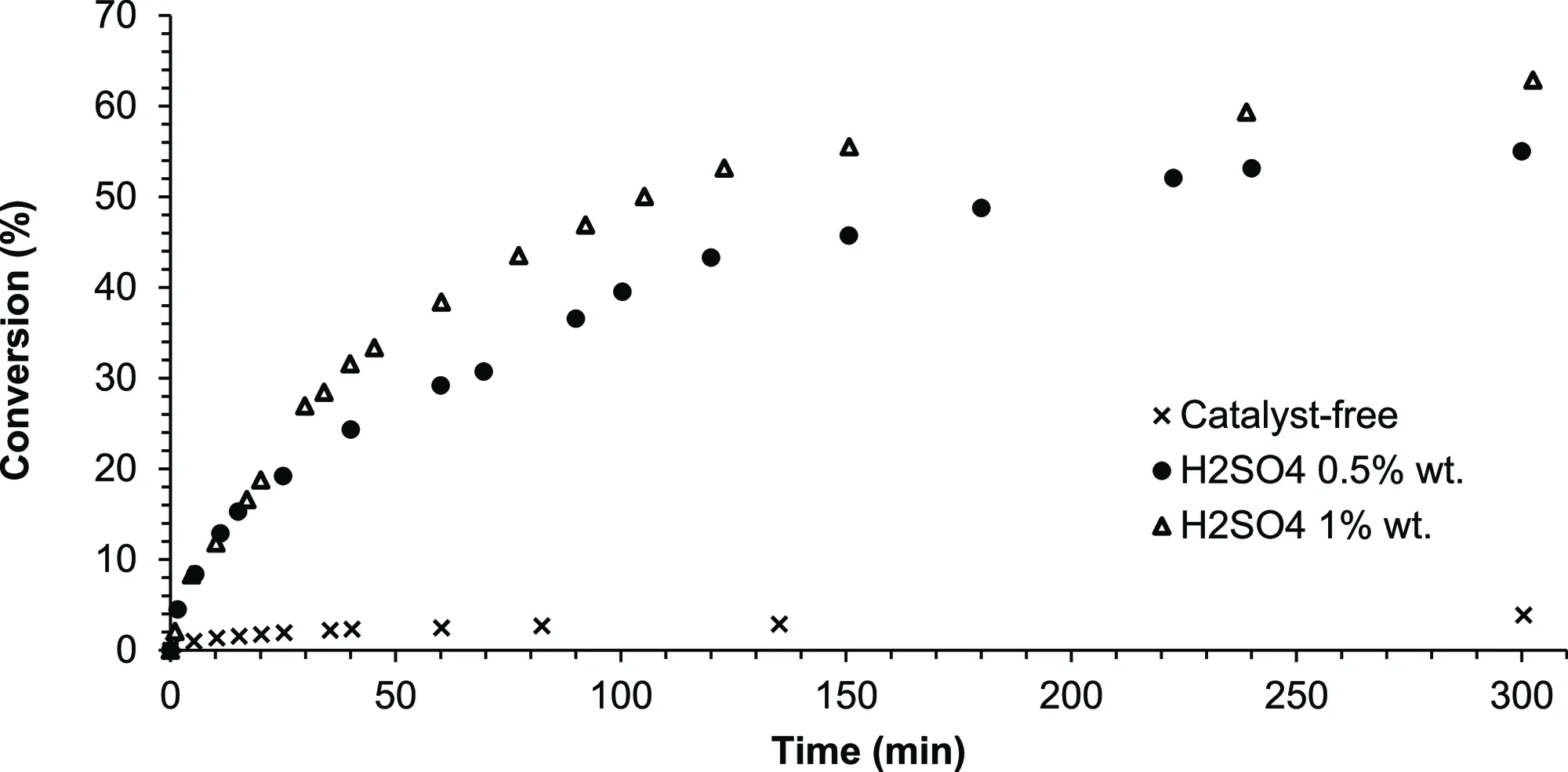
Effect of H_2_SO_4_ catalyst loading on the esterification of stearic acid (SA) with isopropyl alcohol (IPA) (*T* = 70 °C, molar ratio 1:2 SA/IPA).

To obtain a quantitative indicator of catalytic performance, initial reaction rates were calculated at time zero and normalized to the acid equivalents of the catalyst. This analysis showed an increase in turnover frequency (TOF) from 0.49 to 0.79 mol·s^–1^·H^+^ Equiv^1–^ when the H_2_SO_4_ loading was increased from 0.5 to 1 wt %; these values were nearly 2 orders of magnitude higher than those observed for the self-catalyzed reaction. This trend is consistent with that reported for the synthesis of isopropyl myristate,^5^ indicating that the contribution of self-catalysis can be neglected at the evaluated temperatures when strong homogeneous acids are used. Although higher catalyst loadings could be employed, industrial fatty acid esterification is typically conducted with H_2_SO_4_ concentrations up to 1 wt % to minimize product darkening and equipment corrosion.^83^ Therefore, all subsequent experiments with homogeneous and heterogeneous catalysts were performed using acid equivalents corresponding to a 1 wt % H_2_SO_4_ loading. The required amount of each solid catalyst was calculated based on their ion-exchange capacities reported in Table 4. The corresponding kinetic profiles obtained with the commercial catalysts are presented in Figure 6.

**Figure 6:**
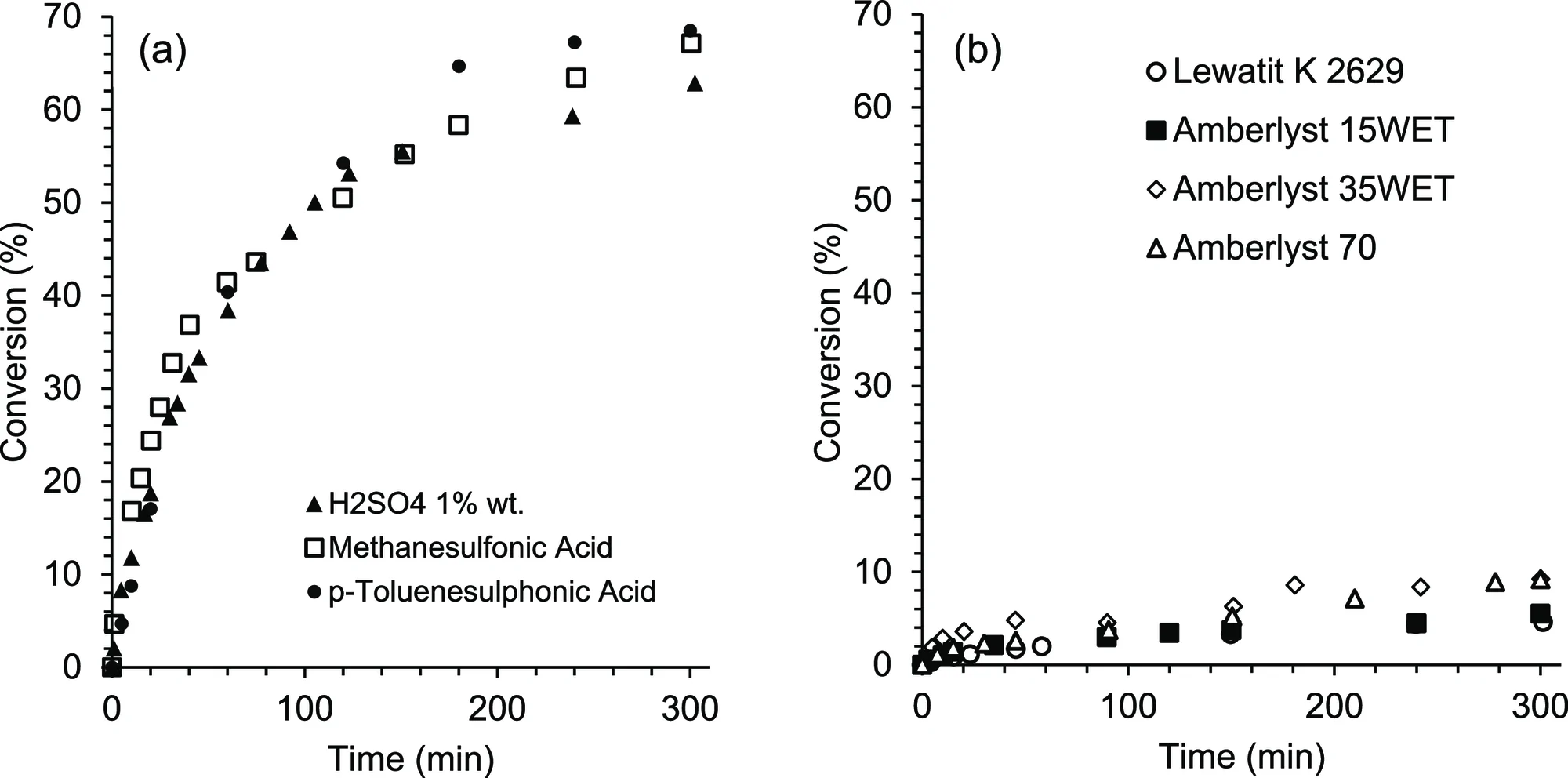
Kinetic profiles during esterification of SA with isopropanol (IPA) (*T* = 70 °C, molar ratio 1:2 SA/IPA) using traditional homogeneous (a) and heterogeneous (b) catalysts. Catalyst loading corresponding to acid equivalents of H_2_SO_4_ at 1 wt % with respect to fatty acids.

As expected, all homogeneous catalysts exhibited higher reaction rates and conversions. Notably, methanesulfonic acid (MSA) achieved comparable, and slightly higher, conversions than H_2_SO_4_, while inducing lower color degradation in the final product. This behavior may be attributed to the lower affinity of H_2_SO_4_ for the organic reaction medium, which could limit dissociation of its second proton and thus reduce its effective catalytic activity. In contrast, MSA and *p*-toluenesulfonic acid possess organic moieties with greater compatibility with the reaction medium (i.e., fatty acid, alcohol, and FAE), enhancing the availability of their single acidic proton, particularly at longer reaction times. The corresponding TOF values, calculated from the initial reaction rate, were 0.47 and 0.49 mol·s^–1^·H^+^ Equiv^1–^ for MSA and *p*-toluenesulfonic acid, respectively. Despite the high catalytic activity, the obtained conversion is below the reported equilibrium conversion (∼93%) at the same conditions here evaluated. This was reached using an enzymatic catalyst.^82^ As lipases require some water to maintain their activity, they are highly effective even after some water is produced during reaction. Additionally, it is important to consider that the reactive mixture can form two liquid phases because stearic acid and isopropyl stearate are immiscible in the produced water.^80^ This can hinder higher conversions using strong sulfonic acids because they are highly water-soluble, thus reducing their catalytic activity in the stearic acid rich phase along reaction.^80–82^ This liquid–liquid behavior can also affect reaction performance when using those heterogeneous catalysts that exhibit water swelling or water retention.

In comparison to homogeneous catalysts, the commercial macroreticular ion-exchange resins exhibited very low catalytic activity. All resins provided only marginally higher conversions than the self-catalyzed reaction, remaining below 10% after 5 h, with apparent TOF values below 0.05 mol·s^–1^·H^+^ Equiv^1–^. This is assumed as apparent as some active sites in micropores of the structure might not be accessible to fatty acids. This trend has been previously reported for fatty acid esterification reactions.^5,10,78,80^ In line with those studies, pore diffusion limitations are unlikely to be the primary cause, given that pore sizes are within the mesoporous range. Instead, the low activity is more likely due to preferential adsorption of isopropanol and water within the resin matrix, which hinders access of fatty acids to the sulfonic acid sites. In addition, swelling and partial shrinking of the polymeric network upon contact with organic species (i.e., fatty acids and esters) may further introduce steric constraints, limiting adsorption and desorption of long-chain fatty acids at the active sites.

Regarding the activity of the Starbon-type catalysts, as shown in Figure 7, both materials exhibited significantly higher performance than the commercial ion-exchange resins, although still lower than that of the homogeneous catalysts. This enhanced performance relative to the resins is consistent with the higher accessible surface area of Starbons, which is considerably greater than that of macroporous commercial resins. In addition, the hydrophobic nature of the carbon framework limits excessive water accumulation within the porous structure and at active sites, in contrast to what is typically observed in macroreticular acid resins. This feature helps mitigate equilibrium limitations in the starch-based catalysts microenvironment. Based on the initial activity, the sulfonated cassava-based Starbon (CS450–SO_3_H) exhibited higher activity than the conventional corn-based material. However, at longer reaction times (∼5 h), S450–SO_3_H outperformed CS450–SO_3_H, reaching conversions of 30% and 24%, respectively. The lower initial rate observed for S450–SO_3_H may be related to its larger particle size (see Figure 3), whereas its superior performance at extended reaction times is likely associated with its larger pore size distribution. The higher fraction of meso- and macropores facilitates both diffusion of fatty acid molecules into the catalyst and desorption of FAEs from the active sites. Also, this could be related with a higher water adsorption in the case of CS450–SO_3_H, which may hinder esterification. It was previously stated that water accumulation could create microenvironments with phase splitting where the fatty acid cannot reach the active sites.^12,55^ The apparent TOF values, based on initial reaction rates, were 0.08 mol·s^–1^·H^+^ Equiv^1–^ for S450–SO_3_H and 0.20 mol·s^–1^·H^+^ Equiv^1–^ for CS450–SO_3_H. Overall, these results suggest that while CS450–SO_3_H shows promising intrinsic activity and may be further evaluated for acid-catalyzed reactions in nonaqueous media (e.g., alkylation, aldol condensation, transesterification), further optimization of pore size distribution and improvement of structural stability are required. Once achieved, additional tests must be conducted in the future to quantitatively assess the effect of water adsorption on the improved catalyst.

**Figure 7:**
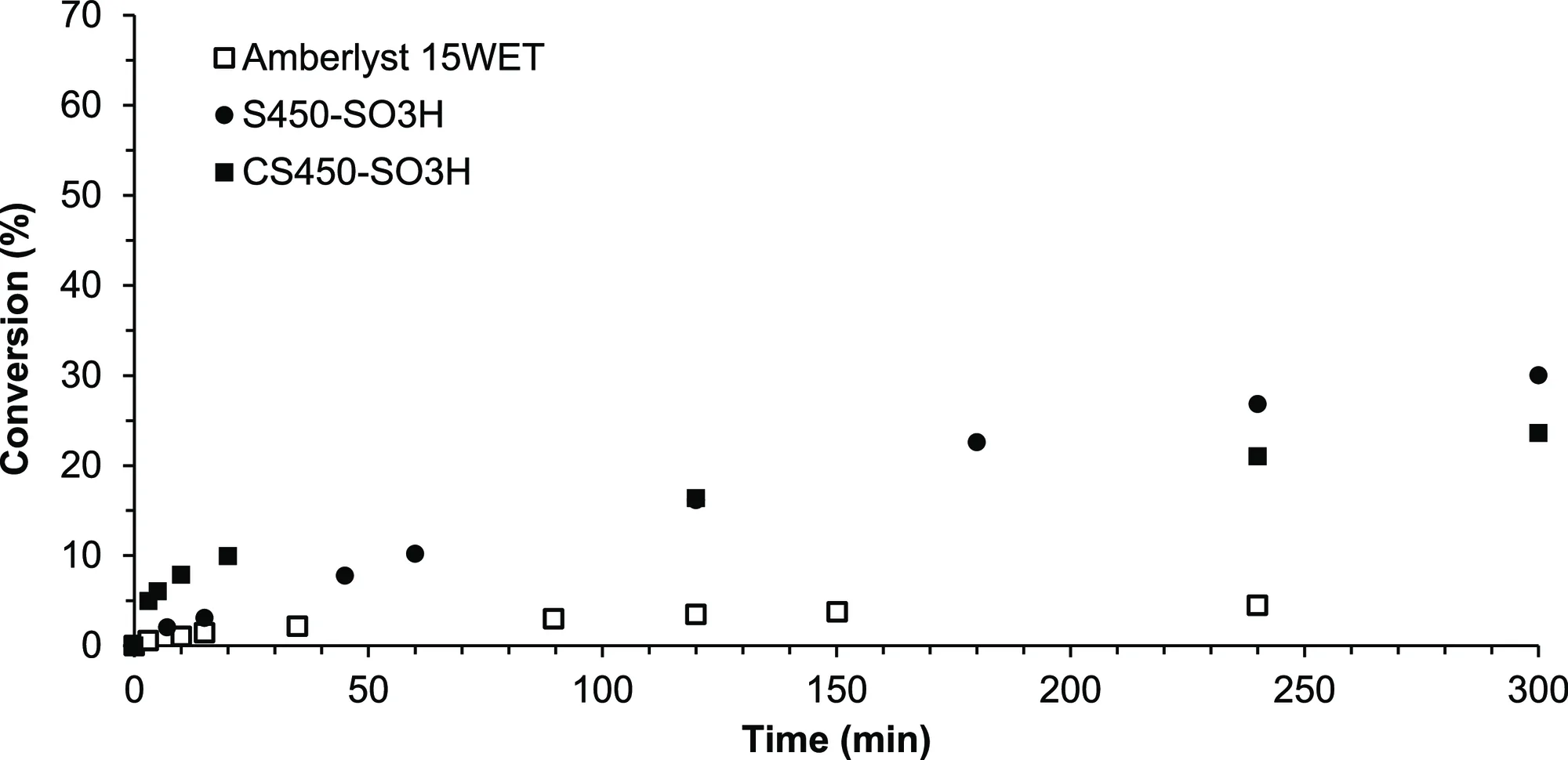
Kinetic profiles during esterification of SA with isopropanol (IPA) (*T* = 70 °C, molar ratio 1:2 SA/IPA) using corn- (S450-SO_3_H) and cassava-based (CS450-SO_3_H) catalysts. Kinetic profile using Amberlyst 15WET is presented as a reference.

The TGA results of the sulfonated materials are presented in Figure 8. Both S450–SO_3_H and CS450–SO_3_H exhibited an initial mass loss step between room temperature and 100 °C, attributed to moisture removal (11.0 and 14.4 wt %, respectively). This behavior is relevant, as it directly reflects the capacity of the materials to retain water within their porous structure during esterification. Although these values are considerably lower than those typically observed for ion-exchange resins after swelling (>60 wt %), and despite the low molecular weight of water, this retained moisture may still contribute to equilibrium limitations. This effect likely contributes to the lower performance of the solid catalysts compared with homogeneous systems and suggests that both Starbon materials may be more suitable for acid-catalyzed reactions under low-water or water-free conditions. Also, this might explain why CS450–SO_3_H exhibited higher initial reaction rates but reached lower conversions compared with S450–SO_3_H at long reaction times.

**Figure 8:**
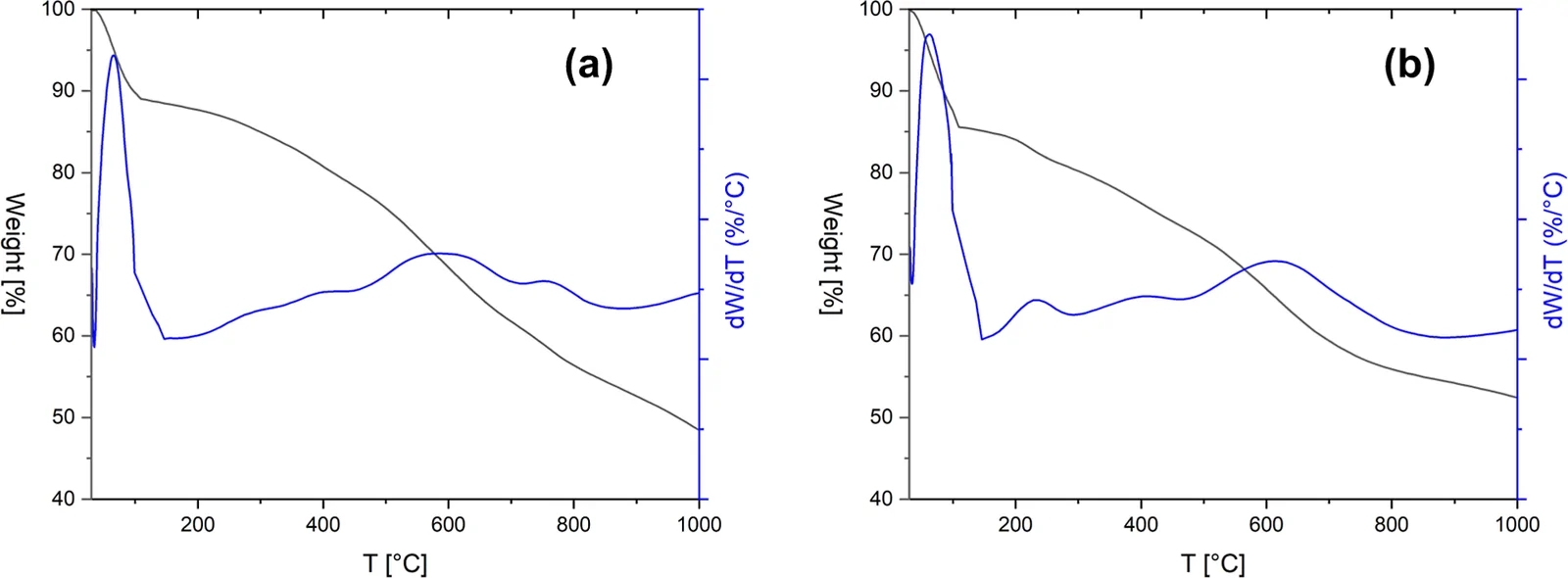
TGA and DTG curves for S450-SO3H (A) and CS450-SO3H (B).

After water removal, the mass of both samples remained essentially constant up to approximately 200 °C. In the temperature range of 200–1000 °C, the observed weight loss is associated with decomposition of surface organic functionalities and progressive aromatization of the carbon structure. At 1000 °C, a significant conversion into a char residue is observed. In particular, S450–SO_3_H retained 56.81 wt % of its initial dry mass, while CS450–SO_3_H retained 55.13 wt %. These results indicate that, to preserve the integrity of sulfonic groups as well as the hydrophilic–hydrophobic balance and mechanical structure of the materials, these catalysts should be operated at temperatures below 200 °C.

Finally, to assess catalysts’ stability of the Starbon-type catalysts, the acidity of the sulfonated materials was measured after the esterification reaction. A slight decrease in acidity was observed, reaching 2.26 eqiuv H^+^·kg^–1^ for S450–SO_3_H (a 2.25% reduction) and 3.49 eqiuv H^+^·kg^–1^ for CS450–SO_3_H (a 3.11% reduction). These small variations indicate that the acidity per unit mass of the materials remains relatively stable. However, only the corn-based material (S450–SO_3_H) could be potentially suitable for reuse, as it was more easily recovered from the reaction medium. In contrast, the reaction mixture containing CS450–SO_3_H became slightly dark, and the recovered catalyst mass was lower than initially loaded, indicating that partial attrition occurred during the reaction. This suggests that future work should focus on evaluating the casava-based catalyst in multiple cycles after improving the mechanical stability of the cassava-derived material. This could be achieved via optimization of the starch carbonization process^69,70^ or by cross-linking with additives (e.g., glutaraldehyde, citric acid) prior to carbonization to improve mechanical strength, also enhancing sulfonation efficiency and acid sites accessibility.^71–75^ To help overcome the negative impacts of water retention, such structural optimization can be also done considering the hydrophobicity/hydrophilicity balance of the surface involving characterization of contact angles.^84–87^ Nevertheless, these results demonstrate, for the first time, the feasibility of producing functional sulfonated cassava-based Starbon catalysts.

## 4 Conclusions

A novel Starbon-type catalyst was successfully synthesized from cassava starch through gelation, retrogradation, solvent exchange, drying, and carbonization, followed by sulfonation for evaluation as an esterification catalyst. The resulting material (CS450–SO_3_H) exhibited a highly mesoporous structure, with an average pore diameter above 8 nm and a BET surface area of 263 m^2^ g^–1^. Its textural properties were broadly comparable to those of a conventional corn-based Starbon (S450–SO_3_H) prepared from a high-amylose commercial starch (Hylon VII). However, the cassava-derived Starbon showed a lower pore volume (0.22 cm^3^ g^–1^) and a higher proportion of micropores (25%), which can be attributed to its higher amylopectin content (∼80%). SEM analysis confirmed that S450–SO_3_H retained the granular mesoporous structure of the gelled starch, whereas CS450–SO_3_H exhibited a collapsed morphology. This structural difference is consistent with the lower amylose content, which reduces the stability of the gel network during solvent exchange, drying, and carbonization. Despite this, sulfonation was more effective for the cassava-derived material, yielding an ion-exchange capacity of 3.60 equiv H^+^·kg^–1^, higher than that of the corn-based catalyst (2.31 eqiuv H^+^·kg^–1^).

In the esterification of stearic acid with isopropanol, CS450–SO_3_H displayed higher reaction rates than commercial ion-exchange resins, although it remained less active than homogeneous catalysts. At longer reaction times, however, the corn-based material achieved higher conversions, likely due to its more open pore structure, which facilitates diffusion and desorption of the produced FAEs. Although predominantly hydrophobic, thermal analysis revealed that CS450–SO_3_H retains approximately 14 wt % water, which may hinder esterification by imposing equilibrium limitations. This suggests that the cassava-derived catalyst may be better suited for water-free reaction systems. Thermal stability was also confirmed up to approximately 200 °C, which defines the upper operating limit for catalytic applications. Finally, no significant loss of acidity per unit mass was observed after reaction (≈3%), indicating good chemical stability. However, CS450–SO_3_H exhibited greater mechanical degradation, leading to partial catalyst loss. Although it showed higher apparent TOF values than other heterogeneous catalysts, further optimization in the synthesis of CS450–SO_3_H is required particularly to preserve the gel structure. This could be achieved via optimization of the starch carbonization process or by cross-linking prior to carbonization to improve mechanical strength and the corresponding hydrophobicity/hydrophilicity balance of the surface. Future work may also explore the application of cassava-derived Starbon catalysts in nonaqueous reactions such as alkylation, aldol condensation, and transesterification.
